# Hybrid Metaheuristics for Solving a Fuzzy Single Batch-Processing Machine Scheduling Problem

**DOI:** 10.1155/2014/214615

**Published:** 2014-04-22

**Authors:** S. Molla-Alizadeh-Zavardehi, R. Tavakkoli-Moghaddam, F. Hosseinzadeh Lotfi

**Affiliations:** ^1^Department of Industrial Engineering, Science and Research Branch, Islamic Azad University, Tehran, Iran; ^2^School of Industrial Engineering, College of Engineering, University of Tehran, Tehran, Iran; ^3^Department of Mathematics, Science and Research Branch, Islamic Azad University, Tehran, Iran

## Abstract

This paper deals with a problem of minimizing total weighted tardiness of jobs in a real-world single batch-processing machine (SBPM) scheduling in the presence of fuzzy due date. In this paper, first a fuzzy mixed integer linear programming model is developed. Then, due to the complexity of the problem, which is NP-hard, we design two hybrid metaheuristics called GA-VNS and VNS-SA applying the advantages of genetic algorithm (GA), variable neighborhood search (VNS), and simulated annealing (SA) frameworks. Besides, we propose three fuzzy earliest due date heuristics to solve the given problem. Through computational experiments with several random test problems, a robust calibration is applied on the parameters. Finally, computational results on different-scale test problems are presented to compare the proposed algorithms.

## 1. Introduction

A batch-processing machine (BPM) is a special variant of a scheduling problem, in which several jobs can be simultaneously processed in such a way that all the jobs in a batch start and complete their processing at the same time. The main advantage is to reduce setups and/or facilitation of material handling. The problem of BPM scheduling is often encountered in real industries. The industrial application of these machines can be found in semiconductor burn-in operations, environmental stress-screening (ESS) chambers, chemical, food, and mineral processing, pharmaceutical and construction materials industries, and so forth.

The BPM scheduling problem is important because the scheduling of batching operations has a significant economic impact. It is mainly motivated by an industrial application, namely, the burn-in operation found in the final testing phase in semiconductor manufacturing [1, 2]. In the semiconductor manufacturing, the jobs have different processing times and sizes that are both required by the customers. The jobs are grouped in batches where a batch means a subset of jobs. The BPM can process a batch of jobs as long as the sum of all the job sizes in the batch does not violate the capacity of the machine. The processing time of a batch is equal to the longest processing time of all the jobs in that batch.

Ikura and Gimple [3] were the first researchers who studied the BPM problem and Lee et al. [4] first presented a detailed description for burn-in operation. As reported in the studies, the exact algorithms have a slow convergence rate and they can solve only small instances to optimality.

As this study addresses SBPM with fuzzy due dates using metaheuristics, the review on SBPM scheduling under a fuzzy environment and the application of metaheuristics to these problems is carried out. For an extensive review on BPM scheduling problems, we refer to Potts and Kovalyov [5] and Mathirajan and Sivakumar [6].

In BPM scheduling problems, Wang and Uzsoy [7] firstly proposed a metaheuristic algorithm. Considering dynamic job arrivals, they combined a dynamic programming algorithm with a random key genetic algorithm (GA) to minimize the maximum lateness. Melouk et al. [8] used a simulated annealing (SA) to minimize the makespan. Koh et al. [9] proposed a random key representation-based GA for the problems of minimizing the makespan and total weighted completion time. Sevaux and Dauzère-Pérès [10], Husseinzadeh Kashan et al. [11], and Damodaran et al. [12] used a GA and redesigned the coding and decoding methods.

Mönch et al. [13] presented a GA combined with dominance properties to minimize the earliness tardiness of the jobs. Chou et al. [14] and Wang et al. [15] presented a hybrid GA and a hybrid forward/backward approach to minimize the makespan. Kashan and Karimi [16] developed two versions of an ant colony optimization (ACO) framework under the situation considered in Koh et al. [9]. Chou and Wang [17], Mathirajan et al. [18], and Wang [19] proposed a hybrid GA, SA, and iterated heuristic for the objective of the total weighted tardiness, respectively. Husseinzadeh Kashan et al. [20] considered bicriteria scheduling for the simultaneous minimization of the makespan and maximum tardiness.

In the classic scheduling problems, it is usually assumed that the aspects of the problem in hand are certain. Most existing models neglect the presence of uncertainty within a scheduling environment. In many real-world scheduling problems, however, uncertainty and vagueness in due date often do exist that make the models more complex. This uncertainty may come about because of production problems (e.g., defect in raw material and machine malfunctioning) or problems with delivery itself (e.g., transportation delay and traffic jam). Although classic BPM scheduling models are extensively studied in the literature, there are only three studies on fuzzy-based BPM models.

Ishii et al. [21] introduced the concept of fuzzy due dates to scheduling problems; fuzzy due dates scheduling problems have been investigated by many researchers. Harikrishnan and Ishii [22] presented a polynomial time algorithm for bicriteria scheduling of serial-batching problem with fuzzy due dates to minimize the total weighted resource consumption and maximize the minimal satisfaction degree. Yimer and Demirli [23] considered a fuzzy goal programming problem for batch scheduling of jobs in a two-stage flow shop to minimize the total weighted flow time of jobs. Cheng et al. [24] proposed ACO to minimize the fuzzy makespan on an SBPM with triangular fuzzy processing times.

Till now, none has considered the objective of minimizing the fuzzy total weighted fuzzy tardiness penalties. So, a new approach to solve a fuzzy SBPM (FSBPM) is proposed and a related fuzzy number is considered for due dates and modeled by fuzzy sets, in which the corresponding membership functions represent satisfaction degree with respect to jobs' completion times. Hence, for the first time, we present a new programming approach. Since the problem is NP-hard for solving the addressed problem, two hybrid metaheuristics (GA-VNS and VNS-SA) are developed to obtain better results.

The remainder of this paper is as follows.  Section 2 describes the problem in detail and presents the fuzzy mathematical model.  Section 3 explains the proposed algorithms.  Section 4 describes the experimental design and compares the computational results. Finally, conclusions are provided and some areas of further research are then suggested in Section 5.

## 2. Fuzzy Mathematical Model and Problem Descriptions

### 2.1. Deterministic Model

The objective of this problem is to minimize the total weighted tardiness penalties. Suppose that there are *n* jobs to be processed and each job *j* ∈ *J* has a processing time *p*_*j*_ and a corresponding size *s*_*j*_. The total size of all the jobs in a batch does not exceed machine capacity *S*. The processing time of a batch *b* is given by the longest job in the batch (i.e., *P* ^*b*^ = max⁡{*p*_*j*_ | *j* ∈ batch  *b*}). The formulation is as follows. 

*Notations*

*Sets*

  
*J*: Jobs, *j* ∈ *J*
 
*B*: Batches, *b* ∈ *B*.

*Parameters*

 
*p*_*j*_: Processing time of job *j*
 
*s*_*j*_: Size of job *j*
 cap: Machine capacity
 
*β*_*j*_: Tardiness penalty (/unit/h) of job *j*
 
*d*_*j*_: Due date of job *j*.

*Decision Variables*

 
*X*_*jb*_: A binary variable indicates the assignment of job *j* to batch *b*
 
*P* ^*b*^: Processing time of batch *b*
 
*c*_*j*_: Completion time of job *j*
 
*C* ^*b*^: Completion time of batch *b*
 
*T*_*j*_: Tardiness of job *j*.

According to the mentioned sets, parameters, and decision variables, the mathematical formulation of the total weighted tardiness penalties can be written below:

(1)  Min⁡ Z=∑j∈JβjTjsj

(2)  s.t:  ∑b∈BXjb=1 ∀j∈J

(3)     ∑j∈JsjXjb≤cap ∀b∈B

(4)     Pb≥pjXjb ∀j∈J,  ∀b∈B

(5)     Cb=∑i=1bPi ∀b∈B

(6)     cj≥Cb−M(1−Xjb) ∀j∈J,  ∀b∈B;

(7)     Tj≥cj−dj ∀j∈J

(8)     Xjb∈{0,1} ∀j∈J,  ∀b∈B.

The objective function is to minimize the total weighted tardiness penalties of jobs. Constraint set (2) ensures that each job can be processed in only one batch. Constraint set (3) ensures that the machine capacity is not exceeded when jobs are assigned to a batch. Constraint set (4) states that the processing time of a batch is the longest processing time among all the jobs in that batch. Constraint set (5) determines the completion time of each batch. Constraint set (6) defines the completion time of each job as the completion time of the batch that it is processed in. Constraint set (7) defines the tardiness of a job as the difference between the due date of a job and its completion time or 0 if it is negative. Constraint set (8) specifies the type of decision variable *X*_*jb*_.

Due to minimization of just only tardiness or total weighted tardiness penalties in the objective function, the model chooses the minimum *P* ^*b*^ in the constraint sets (4) to reach the longest processing time among all the jobs in that batch. The smaller the completion time of jobs, the more desirable the objective function. Similarly, the model finds the minimum *c*_*j*_ and *T*_*j*_ in the constraint sets (6) and (7).

### 2.2. Fuzzy Model

We briefly introduce some basic concepts and results about fuzzy measure theory.

**Definition 1**

If *X* is a collection of objects denoted generically by *x*, then a fuzzy set in *X* is a set of the ordered pairs:

(9) $$\begin{array}{c} \tilde{d}=\left\{x,\tilde{d}\left(x\right)\;|\;x\in X\right\}, \end{array}$$

where $\tilde{d}(x)$ is called the membership function that is associated with each *x* ∈ *X* a number in [0, 1] indicating to what degree *x* is a number.

**Definition 2**

**Figure 1:**
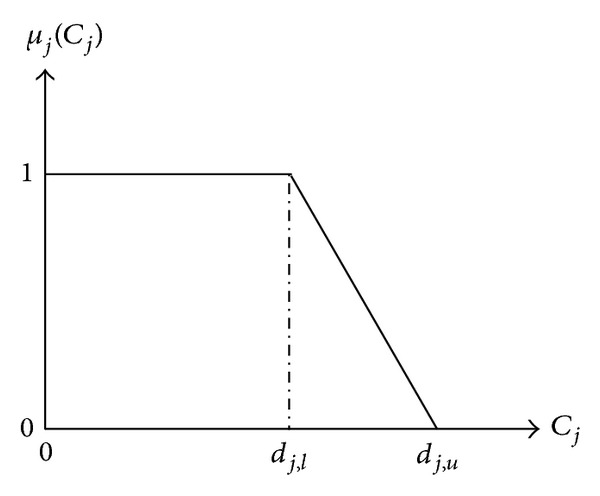
Membership function.

${\tilde{d}}_{j}=\{d_{j,l},d_{j,u}\}$ denotes a fuzzy number as shown in Figure 1.

As mentioned in the literature, the concept of fuzzy due dates has been used in scheduling problems. Here, this concept is being firstly utilized in the BPM scheduling problem. In a fuzzy due date, the membership function assigned to each job represents the customer satisfaction degree for the delivery or completion time of that job. The membership function of a fuzzy due date of a job is represented below:

(10) μj(Cj)={1if  cj≤dj,ldj,u−cjdj,u−dj,lif  dj,l<cj<dj,u0if  cj ≥dj,u.

From Figure 1, we can see that the full satisfaction (i.e., *μ*_*j*_(*C*_*j*_) = 1) is attained if *c*_*j*_ ≤ *d*_*j*,*l*_, and the satisfaction grade is positive if *d*_*j*,*l*_ < *c*_*j*_ < *d*_*j*,*u*_ in the membership function (8). If *d*_*j*,*l*_ = *d*_*j*,*u*_, the fuzzy due date is transformed to interval due date or due window.

According to the mentioned fuzzy due date, the studied problem can be formulated as a maximization problem of the total degree of satisfaction over given jobs or equivalently a minimization problem of the total degree of dissatisfaction. For the fuzzy mathematical formulation, the objective function (11) and constraint sets (12) and (13) are replaced instead of objective function (1) and constraint set (7) to calculate the total degree of satisfaction:

(11) Max⁡Z=∑j∈Jwjμjsj

(12) μj=1 if  cj≤dj,l

(13) μj=dj,u−cjdj,u−dj,l if  dj,l<cj<dj,u.

We can also use the following objective function (20) to calculate the total degree of satisfaction instead of expressions (11)–(13):

(14) Max⁡Z=∑j∈Jwjsj((max⁡(0,dj,l−cj)dj,l−cj)      +(max⁡(0,cj−dj,l)  cj−dj,l)    ×(max⁡(0,dj,u−cj)dj,u−cj)    ×(dj,u−cjdj,u−dj,l)).

It is clear that max⁡(0, *d*_*j*,*l*_ − *c*_*j*_)/(*d*_*j*,*l*_ − *c*_*j*_) = 1 results in *c*_*j*_ ≤ *d*_*j*,*l*_, while max⁡(0, *c*_*j*_ − *d*_*j*,*l*_)/(*c*_*j*_ − *d*_*j*,*l*_) and max⁡(0, *d*_*j*,*u*_ − *c*_*j*_)/(*d*_*j*,*u*_ − *c*_*j*_) = 1 result in *d*_*j*,*l*_ < *c*_*j*_ < *d*_*j*,*u*_. For more explanation of (max⁡(0, *d*_*j*,*l*_ − *c*_*j*_)/(*d*_*j*,*l*_ − *c*_*j*_)) + (max⁡(0, *c*_*j*_ − *d*_*j*,*l*_)/(*c*_*j*_ − *d*_*j*,*l*_))(max⁡(0, *d*_*j*,*u*_ − *c*_*j*_)/(*d*_*j*,*u*_ − *c*_*j*_))((*d*_*j*,*u*_ − *c*_*j*_)/(*d*_*j*,*u*_ − *d*_*j*,*l*_)), consider a simple example of ${\tilde{d}}_{j}=\{d_{j,l},d_{j,u}\}=\{4,7\}$ with different completion times:

(15) (1)  cj=3⟹(max⁡(0,4−3)4−3)+(max⁡(0,3−4)3−4)×(max⁡(0,7−3)7−3)(7−37−4)=1+0×1×43=1.(2)  cj=5⟹(max⁡(0,4−5)4−5)+(max⁡(0,5−4)5−4)×(max⁡(0,7−5)7−5)(7−57−4)=0+1×1×23=23.(3)  cj=8⟹(max⁡(0,4−8)4−8)+(max⁡(0,8−4)8−4)×(max⁡(0,7−8)7−8)(7−87−4)=0+1×0×−13=0.

As mentioned above, similar to the expressions (11)–(14), expressions (1) and (16)–(18) can be used for the equivalent fuzzy mathematical formulation of the total degree of dissatisfaction as follows:

(16) Tj=cj−dj,ldj,u−dj,l if  dj,l<cj<dj,u

(17) Tj=1 if  cj≥dj,u

(18) Min⁡Z=∑j∈Jβjsj((max⁡(0,cj−dj,l)cj−dj,l)    ×(max⁡(0,dj,u−cj)dj,u−cj)    ×(cj−dj,ldj,u−dj,l)    +(max⁡(0,cj−dj,u)cj−dj,u)).

*Linearization*. Obviously, the proposed fuzzy model is a nonlinear mathematical model because of the conditional expressions in the constraint sets (12), (13), (16), and (17). Also, multiplication of variables and max function in the objective functions (14) and (18) are used. An attempt is made in this part to linearize the fuzzy model via introducing binary variable. Hence, the following constraints should be used instead of nonlinear constraint sets (12) and (13):

(19) dj,u−cj≥M(yj−1) ∀j∈J

(20) $$\begin{array}{c} \mu_{j}\leq \frac{d_{j,u}-c_{j}}{d_{j,u}-d_{j,l}}+M\left({1-y}_{j}\right)\;\forall j\in J \end{array}$$

(21) $$\begin{array}{c} \mu_{j}\leq y_{j}\;\forall j\in J \end{array}$$

(22) $$\begin{array}{c} y_{j}\in \left\{0,1\right\}\;\forall j\in J. \end{array}$$

Similarly, the constraint (22), following objective function and constraints, should be used instead of objective function (1) and nonlinear constraint sets (16) and (17):

(23) Min⁡Z=∑j∈Jβj(Tj,1+Tj,2)sjcj−dj,u≤MTj,1 ∀j∈Jdj,u−cj≤Myj ∀j∈JTj,2≥cj−dj,ldj,u−dj,l−M(1−yj) ∀j∈J.

## 3. Solution Approach

The evolutionary computation community has shown for many years significant interest in optimization problems, in particular in the global optimization of real valued problems, for which exact and analytical methods are not productive. These techniques have shown great promise in several real-world applications [25, 26]. Hence, these methods are often utilized in order to solve the problem in a shorter run time.

### 3.1. Proposed Earliest Due Date Heuristics

In this subsection, we propose three constructive greedy heuristics based on EDD as a well-known heuristic method related to the due date. The details of these proposed heuristics are as follows.

- Calculate the index of jobs to be scheduled.
- Sort jobs in increasing order of their index.
- Apply the first-first (FF) heuristic to group jobs into batches.

Accordingly, the details of these three variants of EDDs are as follows:

*EDD Algorithm*. In this variant, the indexes are equal to the EDD of the respective jobs. The centroid-based distance method is used for ranking fuzzy numbers as follows:

(24) Crisp  due  date  (d) =∫0dlx dx+∫dldux((du−x)/(du−dl))dx∫0dldx+∫dldu((du−x)/(du−dl))dx =13(dl+du−dldudl+du).

The jobs are sorted in increasing order of their crisp due dates. So, the job that has the earliest due date will be allotted first.

*EDDL Algorithm*. Sort jobs in increasing order of their *d*_*l*_.

*EDDU Algorithm*. Sort jobs in increasing order of their *d*_*u*_.

### 3.2. Encoding Scheme and Initialization

As mentioned earlier in the literature, the random key (RK) method is used for solving BPM scheduling problems. To generate a sequence by this method, random real numbers between zero and one are generated for each job. By ascending sorting of the value corresponding to each job, the sequence of job is obtained and then the FF heuristic is applied to group the jobs into the batches. After having a permutation and forming the batches, we can use it to compute the objective function value of this solution.

### 3.3. Hybrid Metaheuristics

Over the last years, considerable research has been conducted in hybrid metaheuristics in the field of optimization. The trade-off between intensification and diversification mechanisms is the main aspect of these algorithms. Generally, metaheuristics can be categorized into two main classes: local search methods and population based methods. Population based methods deal with a set of solutions in every iteration of the algorithm, while local search heuristics only deal with a single solution.

Although local search heuristics only deal with a single solution, it has shown its potential in both exploring and exploiting the promising regions in the search space with high quality solutions such as VNS. On the other hand, the basic scheme of VNS and its extensions requires few and sometimes no parameters. However, it is still prone to inferior solutions due to the limited exploration and exploitation ability.

There are two major approaches to hybridize the VNS with other metaheuristics to improve its performance: hybridizing with a local base metaheuristic and hybridizing with a population based metaheuristic. The first idea is to embed SA into VNS, so that it is replaced with local search, whereas SA in hybrid VNS addresses how to get out of large valleys. Besides, SA acts as the local search method, because it is good at searching the neighborhood of a solution. The three neighborhoods employed are swap, insertion, and inversion.

As one of the most well-known population based methods, genetic algorithm (GA) shows robust performance with various problems. Usually, GA has been proven to be very good at shuffling the solution space or global exploration ability but fail to intensify the search towards promising regions. Nevertheless, GA usually takes more computing efforts to locate the optimal in the region of convergence [27], owing to the lack of local search ability. Therefore, hybridization with local search methods may overcome this weakness and lead to powerful search schemes. So, the second idea is to embed VNS as a local search into GA and may be a likely choice to consider the hybridization of them. In GA, VNS is applied as a local search to a subset of offspring generated by one-point crossover and swap mutation operator to search for better solutions.

## 4. Computational Experiments

### 4.1. Instances

To compare the proposed algorithms, some test problems are needed. In this regard, we generate the required data that can affect the performance of the algorithms including the number of jobs (*n*), range of processing time of jobs (*p*_*j*_), size of jobs (*s*_*j*_), tardiness costs (*β*_*i*_), and due date of jobs (*d*_*j*_). The crisp due dates in Tavakkoli-Moghaddam et al. [28] test problems are generated from a uniform distribution. We use such procedure with some modifications to adapt the procedure for our problem as follows:

(25) P−=∑j=1npjn,B−=∑j=1nsj0.8×cap,BP=B−×P−.

After generating the *BP*, the *d*_*j*,*l*_, and *d*_*j*,*u*_ are generated as explained in Table 1.

### 4.2. Parameter Setting

Because of the dependency of metaheuristic algorithms on the correct selection of parameters and operators, we study the behavior of different parameters of proposed algorithms. The parameters of proposed algorithms are as follows: initial temperature (*T*_0_), number of neighborhood search (*n*_max⁡_), reduction ratio of temperature (*α*) population size (*popsize*), crossover percentage (*p*_*c*_), and mutation probability (*p*_*m*_). Levels of these factors are illustrated in Table 2.

In order to be fair, the stopping criterion for all algorithms is equal to 6 × *n* milliseconds. This criterion is sensitive to the problem size. Using this stopping criterion, searching time increases according to the rise in number of jobs. To yield more reliable information and due to having stochastic nature of algorithms, we tackle each test problem ten times. Because the scale of objective functions in each instance is different, they cannot be used directly. To solve this problem, the relative percentage deviation (RPD) is used for each instance. The RPD is obtained by the following formula:

(26) RPD=Algsol−MinsolMinsol×100,

where Alg_sol_ and Min_sol_ are the obtained objective value and minimum objective value found from both proposed algorithms for each instance, respectively. So, we use the RPD measure in the proposed algorithms.

**Figure 2:**
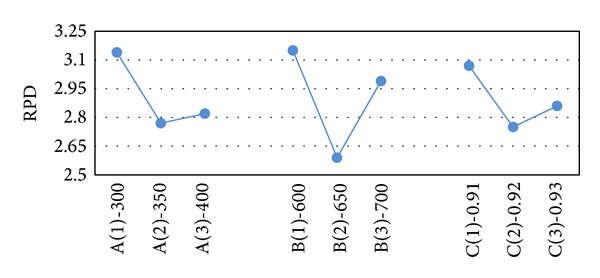
Mean RPD plot for each level of the factors in SA.

**Figure 3:**
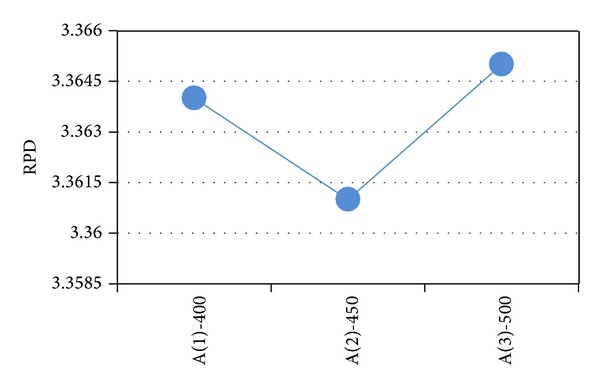
Mean RPD plot for each level of the factors in the VNS.

**Figure 4:**
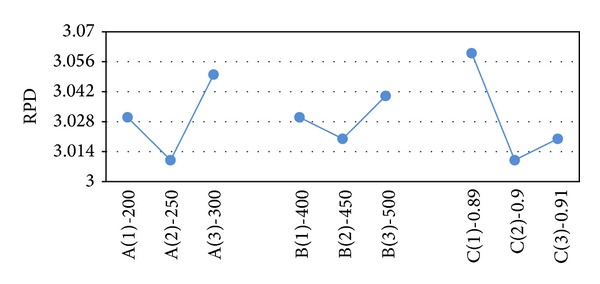
Mean RPD ratio plot for each level of the factors in VNS-SA.

**Figure 5:**
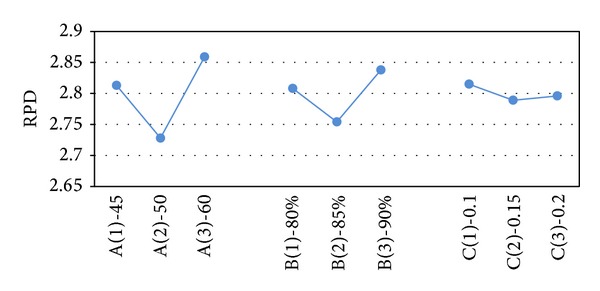
Mean RPD ratio plot for each level of the factors in GA.

**Figure 6:**
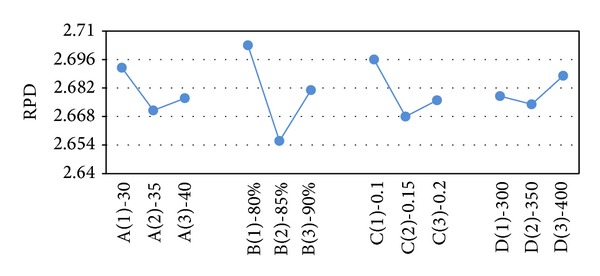
Mean RPD ratio plot for each level of the factors in GA-VNS.

After obtaining the results of the test problems, the results are transformed into RPD measures. The RPD measures are averaged and their value is depicted in Figures 2–6. In SA, better robustness happens when parameters *T*_0_, *n*_max⁡_ and  *α* are 350, 650, and 0.92, respectively, as depicted in Figure 2. In Figure 3, the RPD measure for the single parameter of VNS (*n*_max⁡_) is depicted and the second level or 450 is the best. Also, for hybrid VNS, as illustrated in Figure 4, *T*_0_, *n*_max⁡_, and *α* are defined as 250, 450, and 0.9. In conformity with Figure 5, best magnitude for* popsize*, *p*_*c*_, and *p*_*m*_ in GA are 50, 85%, and 15. Besides, in accordance with Figure 6, in the proposed GA-VNS, best quantity for* popsize*, *p*_*c*_, *p*_*m*_, and *n*_max⁡_ are 35, 85%, 15, and 350, respectively.

### 4.3. Experimental Results

In this section, we present and compare the results of EDDL, EDDU, SA, VNS, GA, VNS-SA, and GA-VNS with the EDD dispatching rule as a well-known heuristic algorithm related to the due date. As mentioned above, we have 60 problem instances, in which each one includes 10 performed replications to achieve the more reliable results.  Table 3 demonstrated the results obtained from EDD, EDDL, and EDDU, in which the first and fourth columns represent the data sets characteristics and the remaining columns show the results on instances.

**Figure 7:**
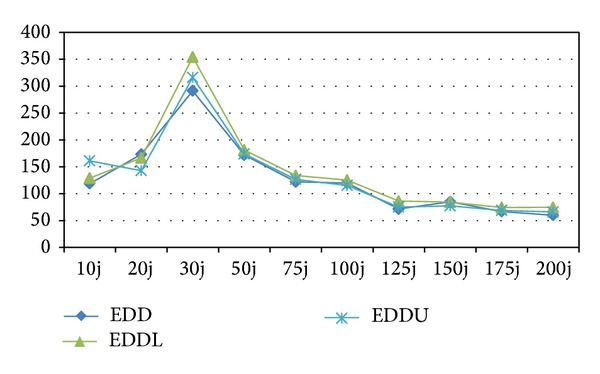
Means plot for the interaction among heuristic algorithms.

**Figure 8:**
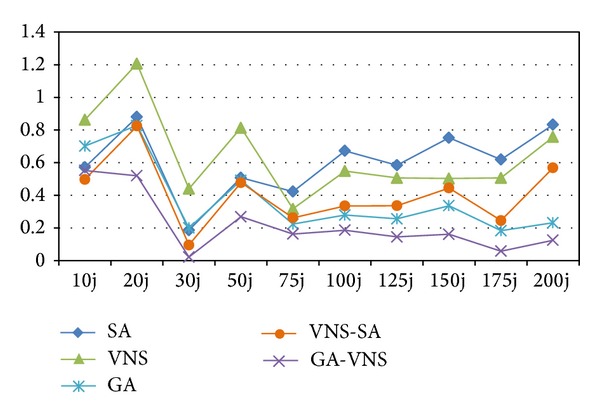
Means plot for the interaction among metaheuristic algorithms.

According to Table 3, among heuristics, EDDL has the worst results, and it can be concluded that EDDU is better than EDD. In order to analyze the interaction between quality of the algorithms and different problem sizes more concisely, the RPD results are calculated for test problems and averaged for each problem size. The average RPDs obtained by each algorithm are shown in Figures 7 and 8. In these figures, each point represents the average results obtained from six test problems considered in each size of problems with ten replications in each algorithm.

It is noticeable that with increasing the problem size, gradually the RPDs of the proposed EDD, EDDL, and EDDU decrease. In spite of decreasing the RPDs, they are not capable to be completive even in the four last sizes.

As it can be seen, the GA-VNS keeps its robust performance in all ranges of problem sizes. On the contrary to SA and VNS, both VNS-SA and GA have good results. VNS-SA has better performance in 10 j and 30 j; however, in the last six problem sizes, GA outperforms it. In the first problem size, SA yields the best result, but with increasing the problem size, gradually its RPDs increase. In the first four problem sizes, VNS does not show a good performance, but with increasing the problem size, its RPDs decrease, while in latter sizes it outperforms SA.

**Figure 9:**
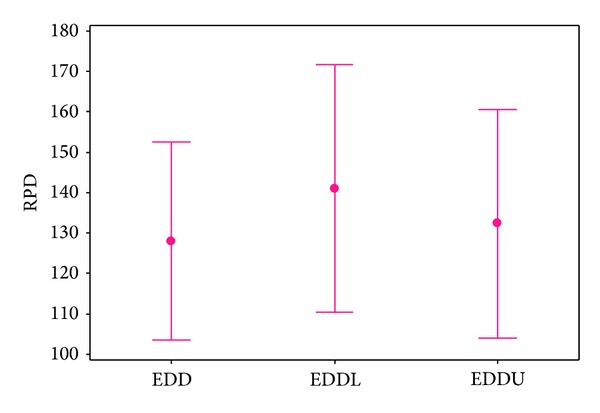
Means plot and LSD intervals for proposed heuristics.

**Figure 10:**
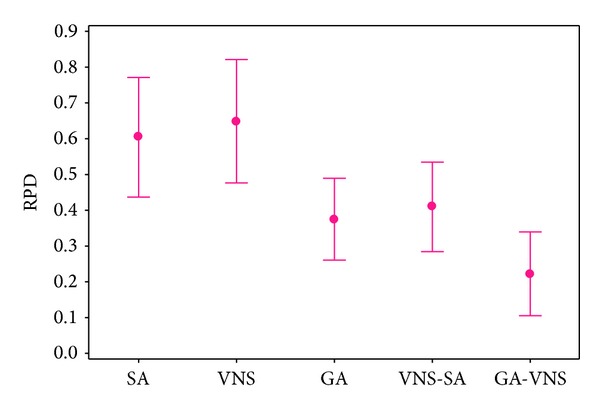
Means plot and LSD intervals for proposed metaheuristics.

From Figures 7 and 8, there is no significant difference between proposed EDD and EDDU or SA and VNS. So, we perform an analysis of variance (ANOVA) to accurately analyze the results among them. The means plot and LSD intervals (at the 95% confidence level) for the presented algorithms are shown in Figures 9 and 10. According to the results, the average RPD obtained by the proposed EDD, EDDL, and EDDU are 128.04, 141, and 132.33 respectively. So, EDD is better than EDDU and EDDL.

Also, the average RPD obtained by the proposed GA-VNS is 0.22, while for GA, VNS-SA, SA, and VNS are 0.37, 0.41, 0.6, and 0.65, respectively. As is evident, GA-VNS has outperformed other algorithm. As it can be seen, between GA and VNS-SA, and also between SA and VNS, there is not a significant difference. However, they failed to statistically overcome each other. However, based on the results, we conclude that the proposed GA-VNS can be used to effectively solve the problem.

## 5. Conclusions and Future Research Directions

In this paper, we discussed the single batch-processing machine (SBPM) scheduling problem in the presence of fuzzy due date to minimize the total weighted tardiness. We developed a mixed integer linear programming model with the objective functions of the total satisfaction or dissatisfaction degree. To solve this model, three heuristics (EDD, EDDL, and EDDU), three metaheuristics (GA, VNS, and SA), and two hybrid metaheuristics (GA-VNS and VNS-SA) are developed. Also, a plan was developed and utilized to generate test problems in a fuzzy environment. To enhance the performance of the proposed method, the experimental design method was used by setting their parameters. The computational results showed that the hybrid GA-VNS were robust and superior to other proposed algorithms. As a future work, total weighted earliness tardiness can be considered as the objective function and the same proposed algorithms can be developed for it. Another direction is to work on other algorithms, such as Cuckoo Optimization Algorithm [29], Honey Bees Optimization [30], Differential Evolution [31], Cuckoo Search [32], and Firefly Algorithm [25, 26].

## Figures and Tables

**Table 1:** Test problems characteristics.

Parameters	Levels	Count
Number of jobs (*N*)	10, 20, 30, 50, 75, 100, 125, 150, 175 and 200	10
Processing time of jobs (*P*)	Uniform distributions [1, 10], [1, 20]	2
Size of jobs (*S*)	Uniform distributions [1, 10], [2, 4], [4, 8]	3
Tardiness cost (*T*)	[5, 8]	1
*d*_*j*,*u*_	Uniform distributions (0.7 × BP, BP)	1
*d*_*j*,*l*_	Uniform distributions (0.6 × *d*_*j*,*u*_, 0.8 × *d*_*j*,*u*_)	1
Cap	10	1

Total number of problem instances		60

**Table 2:** Factors and their levels.

SA, VNS and VNS-SA				GA and GA-VNS		
Parameters	SA levels	VNS levels	VNS-SA levels	Parameters	GA levels	GA-VNS levels
*T*_0_	A(1)—300	—	A(1)—200	*popsize*	A(1)—45	A(1)—30
	A(2)—350	—	A(2)—250		A(2)—50	A(2)—35
	A(3)—400	—	A(3)—300		A(3)—60	A(3)—40

*n*_max_	B(1)—600	A(1)—400	B(1)—400	*p*_*c*_	B(1)—80%	B(1)—80%
	B(2)—650	A(2)—450	B(2)—450		B(2)—85%	B(2)—85%
	B(3)—700	A(3)—500	B(3)—500		B(3)—90%	B(3)—90%

*α*	C(1)—0.91	—	C(1)—0.89	*p*_*m*_	C(1)—0.1	C(1)—0.1
	C(2)—0.92	—	C(2)—0.9		C(2)—0.15	C(2)—0.15
	C(3)—0.93	—	C(3)—0.91		C(3)—0.2	C(3)—0.2

				*n*_max_		D(1)—300
						D(2)—350
						D(3)—400

**Table 3:** Results of EDD, EDDL, and EDDU on test problems.

Problem	EDD	EDDL	EDDU
10jp1s1	113.9	**101.15**	113.9
10jp1s2	**140.49**	189.19	**140.49**
10jp1s3	123.16	**91.03**	132.06
10jp2s1	**119.33**	130.42	185.23
10jp2s2	**113.42**	114.74	**113.42**
10jp2s3	**155.56**	166.44	185.35
20jp1s1	203.7	208.63	**189.57**
20jp1s2	196.16	190.16	**175.72**
20jp1s3	256.24	204.22	**203.58**
20jp2s1	**120.96**	134.36	122.45
20jp2s2	214.69	193.68	**162.22**
20jp2s3	153.54	172.27	**153.37**
30jp1s1	195.61	**193.39**	198.37
30jp1s2	252.3	267.01	**252.26**
30jp1s3	**488.24**	772.72	569.51
30jp2s1	**191.91**	234.04	197.27
30jp2s2	**351.17**	362.43	379.27
30jp2s3	**396.51**	400.85	410.41
50jp1s1	**539.52**	686.09	476.37
50jp1s2	424.3	443.42	**398.52**
50jp1s3	708.06	682.57	**701.39**
50jp2s1	**559.46**	591.87	665.65
50jp2s2	**410.91**	423.84	412.4
50jp2s3	527.77	**390.2**	565.44
75jp1s1	761.27	707.47	**701.15**
75jp1s2	594.23	635.03	**515.88**
75jp1s3	**682.1**	774.99	716.68
75jp2s1	**896.54**	1085.56	1117.63
75jp2s2	517.24	**507.16**	507.64
75jp2s3	872.84	**852.19**	917.32
100jp1s1	**573.12**	631.37	655.84
100jp1s2	874.41	932.05	**864.1**
100jp1s3	**1015.52**	1032.15	1052.11
100jp2s1	**991.97**	1047	946.9
100jp2s2	810.87	**716.69**	636.58
100jp2s3	1365.03	1402.12	**1342.32**
125jp1s1	1263.82	1602.84	**1236.04**
125jp1s2	978.41	**910.7**	989.08
125jp1s3	1090.05	1125.03	**1088.53**
125jp2s1	**968.01**	1303.24	1148.23
125jp2s2	962.34	**942.8**	947.46
125jp2s3	1261.83	**1250.44**	1254.45
150jp1s1	1523.47	1473.88	**1199.04**
150jp1s2	1185.04	**1079**	1417.27
150jp1s3	1654.88	1730.38	**1579.02**
150jp2s1	992.93	**992.75**	897.83
150jp2s2	1167.75	1279.56	**1143.29**
150jp2s3	1727.18	1684.63	**1676.66**
175jp1s1	1150.47	1261.93	**1025.12**
175jp1s2	**1303.52**	1414.09	1335.53
175jp1s3	1888.85	**1805.08**	2013.34
175jp2s1	**1623.61**	1761.51	1876.28
175jp2s2	1408.78	1437.47	**1406.21**
175jp2s3	1651.22	1699.95	**1520.91**
200jp1s1	1198.46	**1142.2**	1160.17
200jp1s2	**1556.72**	1828.91	1724.02
200jp1s3	2266.57	2261.79	**1935.25**
200jp2s1	**1486.81**	1935.52	1912.32
200jp2s2	**1708.57**	1774.06	1731.21
200jp2s3	1892.47	2006.77	**1885.73**

## References

[1] Uzsoy R, Lee C, Martin-Vega L (1992). A review of production planning and scheduling models in the semiconductor industry. Part I. System characteristics, performance evaluation and production planning. *IIE Transactions*.

[2] Uzsoy R, Lee CY, Martin-Vega LA (1994). Review of production planning and scheduling models in the semiconductor industry part II: shop-floor control. *IIE Transactions*.

[3] Ikura Y, Gimple M (1986). Efficient scheduling algorithms for a single batch processing machine. *Operations Research Letters*.

[4] Lee CY, Uzsoy R, Martin-Vega LA (1992). Efficient algorithms for scheduling semiconductor burn-in operations. *Operations Research*.

[5] Potts CN, Kovalyov MY (2000). Scheduling with batching: a review. *European Journal of Operational Research*.

[6] Mathirajan M, Sivakumar AI (2006). A literature review, classification and simple meta-analysis on scheduling of batch processors in semiconductor. *International Journal of Advanced Manufacturing Technology*.

[7] Wang CS, Uzsoy R (2002). A genetic algorithm to minimize maximum lateness on a batch processing machine. *Computers and Operations Research*.

[8] Melouk S, Damodaran P, Chang P-Y (2004). Minimizing makespan for single machine batch processing with non-identical job sizes using simulated annealing. *International Journal of Production Economics*.

[9] Koh SG, Koo PH, Kim DC, Hur WS (2005). Scheduling a single batch processing machine with arbitrary job sizes and incompatible job families. *International Journal of Production Economics*.

[10] Sevaux M, Dauzère-Pérès S (2003). Genetic algorithms to minimize the weighted number of late jobs on a single machine. *European Journal of Operational Research*.

[11] Husseinzadeh Kashan A, Karimi B, Jolai F (2006). Effective hybrid genetic algorithm for minimizing makespan on a single-batch-processing machine with nonidentical job sizes. *International Journal of Production Research*.

[12] Damodaran P, Kumar Manjeshwar P, Srihari K (2006). Minimizing makespan on a batch-processing machine with non-identical job sizes using genetic algorithms. *International Journal of Production Economics*.

[13] Mönch L, Unbehaun R, Choung YI (2006). Minimizing earliness-tardiness on a single burn-in oven with a common due date and maximum allowable tardiness constraint. *OR Spectrum*.

[14] Chou FD, Chang PC, Wang HM (2006). A hybrid genetic algorithm to minimize makespan for the single batch machine dynamic scheduling problem. *International Journal of Advanced Manufacturing Technology*.

[15] Wang HM, Chang PC, Chou FD (2007). A hybrid forward/backward approach for single batch scheduling problems with non-identical job sizes. *Journal of the Chinese Institute of Industrial Engineers*.

[16] Kashan AH, Karimi B (2008). Scheduling a single batch-processing machine with arbitrary job sizes and incompatible job families: an ant colony framework. *Journal of the Operational Research Society*.

[17] Chou FD, Wang HM (2008). Scheduling for a single semiconductor batch-processing machine to minimize total weighted tardiness. *Journal of the Chinese Institute of Industrial Engineers*.

[18] Mathirajan M, Bhargav V, Ramachandran V (2010). Minimizing total weighted tardiness on a batch-processing machine with non-agreeable release times and due dates. *International Journal of Advanced Manufacturing Technology*.

[19] Wang HM (2011). Solving single batch-processing machine problems using an iterated heuristic. *International Journal of Production Research*.

[20] Husseinzadeh Kashan A, Karimi B, Jolai F (2010). An effective hybrid multi-objective genetic algorithm for bi-criteria scheduling on a single batch processing machine with non-identical job sizes. *Engineering Applications of Artificial Intelligence*.

[21] Ishii H, Tada M, Masuda T (1992). Two scheduling problems with fuzzy due-dates. *Fuzzy Sets and Systems*.

[22] Harikrishnan KK, Ishii H (2005). Single machine batch scheduling problem with resource dependent setup and processing time in the presence of fuzzy due date. *Fuzzy Optimization and Decision Making*.

[23] Yimer AD, Demirli K (2009). Fuzzy scheduling of job orders in a two-stage flowshop with batch-processing machines. *International Journal of Approximate Reasoning*.

[24] Cheng B, Li K, Chen B (2010). Scheduling a single batch-processing machine with non-identical job sizes in fuzzy environment using an improved ant colony optimization. *Journal of Manufacturing Systems*.

[25] Zhang Y, Wang S, Ji G, Dong Z (2013). Genetic pattern search and its application to brain image classification. *Mathematical Problems in Engineering*.

[26] Zhang Y, Wu L, Wang S (2013). Solving two-dimensional HP model by firefly algorithm and simplified energy function. *The Scientific World Journal*.

[27] Wu AS, Yu H, Jin S, Lin K-C, Schiavone G (2004). An incremental genetic algorithm approach to multiprocessor scheduling. *IEEE Transactions on Parallel and Distributed Systems*.

[28] Tavakkoli-Moghaddam R, Rahimi-Vahed A, Mirzaei AH (2007). A hybrid multi-objective immune algorithm for a flow shop scheduling problem with bi-objectives: weighted mean completion time and weighted mean tardiness. *Information Sciences*.

[29] Balochian S, Ebrahimi E (2013). Parameter optimization via cuckoo optimization algorithm of fuzzy controller for liquid level control. *Journal of Engineering*.

[30] Celik Y, Ulker E (2013). An improved marriage in honey bees optimization algorithm for single objective unconstrained optimization. *The Scientific World Journal*.

[31] Choi TJ, Ahn CW, An J (2013). An adaptive cauchy differential evolution algorithm for global numerical optimization. *The Scientific World Journal*.

[32] Zhou Y, Zheng H (2013). A novel complex valued cuckoo search algorithm. *The Scientific World Journal*.

